# PSYCHOMETRIC PROPERTIES OF THE TIMED BACKWARD WALK TEST IN PEOPLE WITH STROKE

**DOI:** 10.2340/jrm.v58.46292

**Published:** 2026-10-12

**Authors:** Yu QIN, Siyue LI, Peiming CHEN, Thomson W.L. WONG, Cynthia Y.Y. LAI, Shamay S.M. NG

**Affiliations:** Department of Rehabilitation Sciences, The Hong Kong Polytechnic University, Hong Kong SAR, China

**Keywords:** gait, postural balance, psychometrics, rehabilitation, stroke, walking

## Abstract

**Objective:**

To examine the psychometric properties of the Timed Backward Walk test in people with stroke.

**Design:**

Cross-sectional study.

**Subjects:**

60 people with stroke and 30 healthy adults aged 55 years and older.

**Methods:**

Participants performed the Timed Backward Walk test and standard clinical assessments. Discriminative validity was evaluated using receiver operating characteristic curve. Reliability (one-week test–retest, inter-rater, intra-rater) was assessed using intraclass correlation coefficients. Construct validity was examined through correlations with motor function, strength, balance, and gait measures.

**Results:**

People with chronic stroke took significantly longer to complete the test than healthy adults (*p* < 0.001), with an area under the curve of 0.968 and an optimal cut-off of 7.22 s. Test–retest, inter-rater, and intra-rater reliability were all excellent. The standard error of measurement was 1.62 s and the minimal detectable change was 4.49 s. The complete time showed strong positive correlations with functional mobility and gait speed measures (ρ = 0.840–0.897) and moderate negative correlations with balance, lower-limb motor function, and ankle dorsiflexor strength (ρ = –0.408 to –0.647).

The Timed Backward Walk test demonstrated excellent reliability, discriminative validity, and construct validity in people with chronic stroke. It is a feasible and sensitive complement to forward gait assessments.

## LAY ABSTRACT

After a stroke, many people struggle with walking and balance. Walking backwards is a challenging task that can reveal hidden mobility issues. In this study, we tested a simple assessment: timing how long it takes to walk backwards for a short distance. We compared 60 stroke survivors with 30 healthy adults aged 55 years and older. We found that stroke survivors took longer to complete the task. More importantly, we proved that this simple timing test is highly consistent and trustworthy. We identified a specific target time of about 7 s that clearly separates healthy walking from stroke-affected walking. For everyday medical practice, this is highly valuable. Physical therapists now have a quick and easy tool that requires no special equipment. They can use it to easily check a patient’s balance, monitor their recovery progress over time, and design better rehabilitation programmes to improve their daily independence.

Backward walking is an important yet often overlooked component of functional mobility. In daily life, people often need to step backwards when mopping, opening doors, making way in narrow corridors, or pulling heavy objects. The ability to walk backwards safely is therefore essential for independent living and community mobility (1).

One of the main challenges of backward walking is the limited availability of visual information. Due to limited visual feedback, individuals must rely heavily on proprioception and vestibular feedback to maintain direction and postural control during backward walking (2). Compared with forward walking, it places greater demands on the hip extensors and hamstrings, and requires a higher degree of lower-limb coordination (2, 3). Despite these unique demands, backward walking has received far less attention than forward walking in clinical assessment and rehabilitation research.

Following stroke, impairments such as muscle weakness, abnormal muscle tone, impaired motor control, reduced proprioception, and poor balance often limit locomotor performance (4, 5). Although many individuals regain forward walking ability, backward walking remains particularly challenging because it places heavy demands on proprioceptive processing, hip extensor and hamstring strength, and dynamic postural control. In addition, stroke-related impairments in proprioceptive and vestibular function may further compromise performance during backward walking. Studies have found that backward walking speed is more sensitive to motor and balance deficits than forward walking speed in neurological populations (6, 7), suggesting it may reveal residual impairments not captured by forward walking assessments alone.

Accurate measurement of walking performance is essential in stroke rehabilitation. Most standardized gait and balance assessments focus on forward walking (8). However, because forward walking is a habitual, visually guided task, stroke survivors may use compensatory strategies to maintain speed, such as greater reliance on the non-paretic limb (9–11). These adaptations can mask deficits in proprioception and postural control, reducing assessment sensitivity, especially among higher-functioning individuals (12). Adding backward walking assessments may therefore provide a more comprehensive evaluation of sensorimotor function and balance.

Several clinical tests have been used to assess backward walking after stroke. The 3-Metre Backward Walk Test (3MBWT) has shown excellent intra-rater and inter-rater reliability (ICC = 0.94–0.99) and good concurrent validity with the Berg Balance Scale (*r* = −0.691) and the Timed Up and Go test (*r* = 0.849) (13, 14). However, the short testing distance may be insufficient to accommodate the acceleration and deceleration phases of walking, thereby limiting the opportunity for individuals to transition from gait initiation and acceleration to steady-state walking velocity (15). This may restrict the relevance of the 3MBWT to higher-functioning, community-dwelling individuals. In contrast, the 10 m Timed Backward Walk (TBW) test may better capture this steady state velocity and thus reflect functional walking capacity more accurately, although its psychometric properties in stroke have not been systematically established.

To address the gap, this study aimed to establish the psychometric properties of the TBW test in people with chronic stroke. The objectives were to: (a) assess known-group validity by comparing TBW performance between people with chronic stroke and healthy adults aged 55 years and older; (b) examine test–retest, intra-rater, and inter-rater reliability and determine the minimal detectable change; and (c) evaluate concurrent validity against established measures of gait, balance, and lower-limb motor function.

## METHODS

### Study design

A methodological cross-sectional study was conducted from February to July 2024. The study design and reporting followed the Strengthening the Reporting of Observational Studies in Epidemiology (STROBE) statement and the COnsensus-based Standards for the selection of health Measurement INstruments (COSMIN) recommendations for studies on measurement properties (16, 17). This study was conducted in accordance with the Declaration of Helsinki and its later amendments, and approved by the PolyU Institutional Review Board of The Hong Kong Polytechnic University (No.: HSEARS20230807001). Informed consent was obtained from all participants prior to their inclusion in the study.

### Sample size calculation

The required sample size for the reliability analysis was estimated *a priori* using an ICC based on a previous study (14). A minimum acceptable reliability of 0.90 and an expected reliability of 0.96 were assumed, with a two‑tailed significance level of 0.05 and a statistical power of 80%, yielding a required sample of 36 participants.

The sample size for the validity analysis was calculated *a priori* using G*Power 3.1.9.7 (Franz Faul, University of Kiel, Germany). A two‑tailed correlation model was specified with an anticipated effect size from another study of |ρ| = 0.691 (13), α = 0.05, and power=0.80, resulting in a required total sample of 11 participants.

Allowing for an anticipated dropout rate of 10%, at least 40 individuals with stroke were required to meet the study objectives. To ensure adequate statistical power and to enhance the precision of validity estimates across diverse clinical presentations, a larger sample than the minimum required was recruited.

### Participants

A total of 60 people with chronic stroke and 30 healthy adults aged 55 years and older were recruited through poster advertisements within community‑based self-help rehabilitation groups.

Eligibility criteria for the stroke group were: (*i*) age ≥ 55 years; (*ii*) clinical diagnosis of a stroke at least 6 months previously; (*iii*) ability to provide written informed consent; (*iv*) ability to complete the assessments independently, with or without walking aids; (*v*) score ≥ 7 on the Abbreviated Mental Test (18); and (*vi*) stable general medical status. Participants with additional neurological disorders, unstable comorbidities, or any medical or rehabilitation interventions during the 1‑week interval between test sessions that could influence balance or mobility (e.g., botulinum toxin injections, physiotherapy, or other intensive rehabilitation programmes) were excluded.

Neurologically healthy adults were recruited as a comparison group. They were required to be aged 55 years or older and in stable general health. Individuals with any history of neurological disease or with unstable cardiovascular conditions that could compromise performance were excluded.

### Procedures

All study procedures were conducted in a university-based rehabilitation laboratory (see Supplementary Fig. S1). Two trained research personnel (QY and LSY), both with health-related training backgrounds, administered the TBW test and all outcome measures.

Participants with chronic stroke attended 2 sessions 1 week apart. At the first session, demographic information, clinical characteristics, and all outcome measures, including the TBW test and other mobility and balance tests, were obtained. To assess inter-rater reliability, research personnel independently and simultaneously timed the TBW test during the first session using separate stopwatches, without observing each other’s recordings. For test–retest reliability, the TBW test was reassessed by the same examiner after a 1-week interval. This interval was chosen to minimize practice or learning effects while maintaining stable balance and mobility status.

Healthy adults only attended a single assessment session, during which demographic data were collected and the TBW test was completed.

### TBW test

The TBW test was conducted in a quiet corridor on a flat, unobstructed surface. A 10-m walkway was marked, with an additional 1-m acceleration distance and a 1-m deceleration distance.

Participants were instructed to walk backwards along the walkway at a comfortable, self-selected speed, using their usual walking aids if required. Timing was recorded with a handheld stopwatch and started when the first backward step crossed the start line and stopped when both feet had crossed the 10-m finish line. Each participant completed 2 consecutive trials, and the mean time (in seconds) of the 2 valid trials was used for analysis. A physiotherapist walked slightly behind and to the side of the participant during each trial to ensure safety.

### Other outcome measures

The Fugl-Meyer Assessment of Lower Extremity (FMA-LE) was used to assess lower-limb motor impairment in people with stroke, including reflex activity, volitional movement with or without synergy, and coordination (19). Seventeen items are scored on a 3-point scale (0–2), giving a total score of 0–34; higher scores indicate better motor recovery. The FMA-LE is widely used in stroke rehabilitation research and clinical practice as a criterion measure of lower limb motor function.

Maximal isometric strength of the ankle muscles was measured with a handheld dynamometer (Model 01160; Lafayette Instrument Company, Lafayette, IN, USA). Participants performed two 5-s maximal contractions, and the mean force (kg) was used for analysis.

Clinical balance performance was evaluated with the Berg Balance Scale (BBS). It consists of 14 items assessing balance during everyday functional tasks scored from 0 to 4, yielding a total score of 0–56; higher scores indicate better balance (20). The BBS is one of the most commonly used clinical balance scales in stroke populations and has been linked to fall risk.

Dynamic postural control during voluntary limits of stability was assessed using a computerized dynamic posturography system (Bertec Corporation, Columbus, OH, USA). This system quantifies the maximum voluntary displacement of the centre of pressure in multiple directions without changing the base of support (21). Outcome variables included reaction time (LOS_RT), movement velocity (LOS_MVL), endpoint excursion (LOS_EPE), maximum excursion (LOS_MXE), and directional control (LOS_DCL).

Sensory organization during quiet stance was evaluated using the Sensory Organization Test (SOT) on the same dynamic posturography system. Participants stood quietly under 6 standardized sensory conditions that systematically altered or removed visual and somatosensory inputs while keeping the base of support constant (22). Sensory ratios were calculated to estimate the effective use of somatosensory (SOM), visual (VIS), and vestibular information (VEST), as well as visual preference (PREF).

Walking speed was measured using the 10-Metre Walk Test (10MWT) over a 10-m walkway with 1-m acceleration and 1-m deceleration zones to capture steady state gait (23). Each participant completed 2 trials at a comfortable speed and 2 trials at their fastest safe speed. The mean time for each condition was used for analysis.

Functional mobility was assessed with the Timed Up and Go (TUG) test. Participants were instructed to stand up from a chair, walk 3 m, turn, walk back, and sit down (24). The time to complete the task was recorded, with shorter times indicating better mobility. The TUG captures multiple components of functional mobility, including sit to stand, gait initiation, turning, and dynamic balance.

Participation and activity were evaluated using the Cantonese version of the Oxford Participation and Activities Questionnaire (Ox-PAQ), a patient-reported outcome measure for individuals with neurological conditions (25). It contains 23 items in 3 domains (routine activities, social engagement, and emotional well-being), each scored from 0 (never) to 4 (always); higher scores reflect greater difficulty. Domain and total scores were used to quantify self-perceived limitations in daily activities and social participation.

### Statistical analysis

All statistical analyses were performed using IBM SPSS Statistics version 26 (IBM Corp, Armonk, NY, USA). Continuous variables were inspected for normality using the Shapiro–Wilk test. Demographic and clinical characteristics are reported as mean (standard deviation, SD) or median (interquartile range, IQR), as appropriate, and categorical variables as counts and percentages. Between-group comparisons were conducted with independent‑samples *t*‑tests or Mann–Whitney *U* tests, depending on data distribution.

Known groups and discriminative validity of the TBW test were examined between people with chronic stroke and healthy adults using independent-samples *t*-tests or Mann–Whitney *U* tests, depending on data distribution. Effect sizes (Cohen’s *d* for parametric tests or *r* for non-parametric tests) were calculated to indicate the magnitude of between-group differences. Receiver operating characteristics (ROC) curve analyses were conducted to evaluate the discriminative ability of the TBW test to distinguish between stroke participants and healthy controls. The area under the curve (AUC) and 95% confidence interval (CI) were calculated, with AUC values of 0.70–0.79, 0.80–0.89, and ≥ 0.90 considered acceptable, excellent, and outstanding discrimination, respectively (26). Optimal TBW cut-off time was identified using Youden’s index (sensitivity + specificity − 1), and the corresponding sensitivity and specificity were reported.

The reliability of the TBW test was examined using ICCs. Test–retest reliability of the TBW test in people with stroke was examined using a two-way mixed-effects ICC with absolute agreement for average measures (ICC_3,2_). Intra-rater reliability was evaluated using a two-way mixed-effects ICC with absolute agreement for single measures (ICC_3,1_). Inter-rater reliability was examined using a two-way random-effects ICC with absolute agreement for average measures (ICC_2,2_). ICC values < 0.50, 0.50–0.75, 0.75–0.90, and > 0.90 were interpreted as poor, moderate, good, and excellent reliability, respectively (27). For test–retest reliability, measurement error was quantified using the standard error of measurement (SEM = SDpooled × √ (1 − ICC)) and the minimal detectable change at the 95% confidence level (MDC_95_ = 1.96 × SEM × √2) (28). A Bland–Altman plot was used to visually inspect systematic bias and limits of agreement between test and retest TBW times.

Construct validity was determined in the stroke group by examining correlations between TBW time and other outcome measures. Pearson’s correlation coefficients were calculated for approximately normally distributed variables; otherwise, Spearman’s rank correlation coefficients were used. Correlations were interpreted as weak (< 0.40), moderate (0.40–0.69), or strong (≥ 0.70) (29).

## RESULTS

### Participant characteristics

A total of 90 participants were recruited, including 60 people with chronic stroke and 30 healthy adults aged 55 years and older. Demographic and clinical characteristics are summarised in Table I. The 2 groups did not differ significantly in age, gender, or body mass index (BMI) (*p* > 0.05). TBW performance is presented in Table II.

**Table I:** Demographic and clinical characteristics of the participants

Parameter	People with chronic stroke (*n* = 60)	Healthy adults aged 55 years and older (*n* = 30)	*p*-value
Age, years, mean (SD)	66.63 (6.08)	63.90 (7.90)	0.103
Sex, M/F, *n* (%)	32 (53.3)/28 (46.7)	17 (56.7)/13 (43.3)	0.768
BMI, kg/m², mean (SD)	23.91 (3.19)	23.69 (4.05)	0.778
Mobility aids, Yes/No, *n* (%)	20 (33.3)/40 (66.7)	/	/
Stroke type, I/H/Both, *n* (%)	36 (60.0)/23 (38.3)/ 1 (1.7)	/	/
Hemiplegic side, L/R, *n* (%)	28 (46.7)/32 (53.3)	/	/
Time since stroke, years, median (IQR)	8.00 (4.80)		

SD: standard deviation; BMI: body mass index; I: ischaemic; H: haemorrhagic; L: left; R: right; IQR: interquartile range.

**Table II:** Timed backward walking (TBW) performance in people with chronic stroke and healthy adults aged 55 years and older

Parameter	People with chronic stroke (*n* = 60)	Healthy adults aged 55 years and older (*n* = 30)	*p*-value
TBW time, s, Day 1, Rater A, median (IQR)	17.38 (14.55)	4.85 (1.83)	< 0.001*
TBW time, s, Day 1, Rater B, median (IQR)	17.22 (14.67)	/	/
TBW time, s, Day 2, median (IQR)	16.40 (17.79)	/	/

IQR: interquartile range.

* Significant difference between the two groups (*p* < 0.001).

### Known groups and discriminative validity

People with chronic stroke took significantly longer TBW time than healthy adults (U = 57.0, Z = –7.216, *p* < 0.001). The discriminative ability of TBW test to distinguish people with chronic stroke from healthy adults was further examined using ROC curve analysis. The AUC for TBW time was 0.968 (95% CI 0.934–1.000, *p* < 0.001; Fig. 1), indicating outstanding discrimination. A cut-off value of 7.22 s for TBW time provided a sensitivity of 96.7% and a specificity of 90.0% for identifying individuals with chronic stroke.

**Fig. 1:**
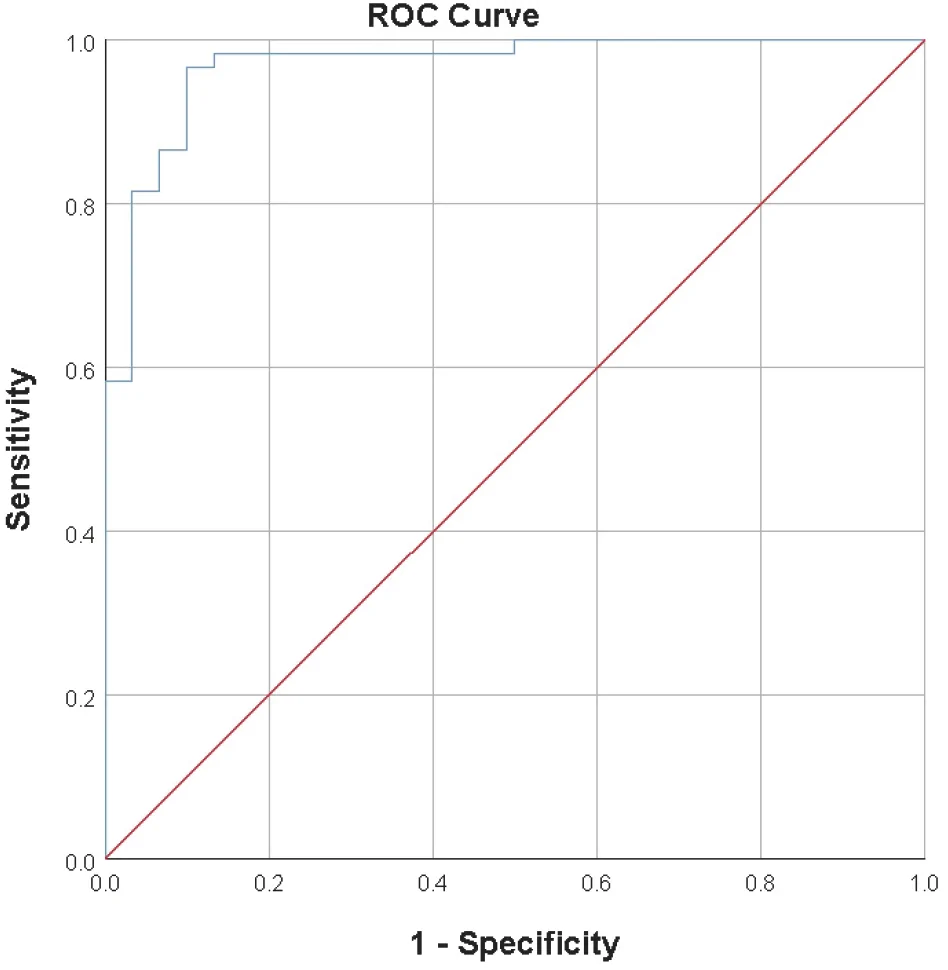
Receiver operating characteristics (ROC) curves of timed backward walking test for discriminating patients from healthy controls.

### Reliability

Overall, the TBW test demonstrated excellent reliability in people with chronic stroke. Excellent reliability was observed for both inter-rater (ICC_2,2_ = 0.999, 95% CI: 0.998–0.999, *p* < 0.001) and intra-rater (ICC_3,1_ = 0.962, 95% CI: 0.937–0.977, *p* < 0.001) assessments. For test–retest reliability, the ICC_3,2_ for TBW time was 0.987 (95% CI 0.979–0.992, *p* < 0.001). Bland–Altman analysis revealed a minimal mean difference (bias) of 0.16 s between the 2 timepoints, with 95% limits of agreement (LoA) ranging from –4.29 s to 4.61 s (Fig. 2). The majority of data points fell within the 95% LoA without exhibiting any obvious proportional bias across the range of mean values. Based on the pooled standard deviation of TBW times, the SEM was 1.62 s, resulting in an MDC_95_ of 4.49 s.

**Fig. 2:**
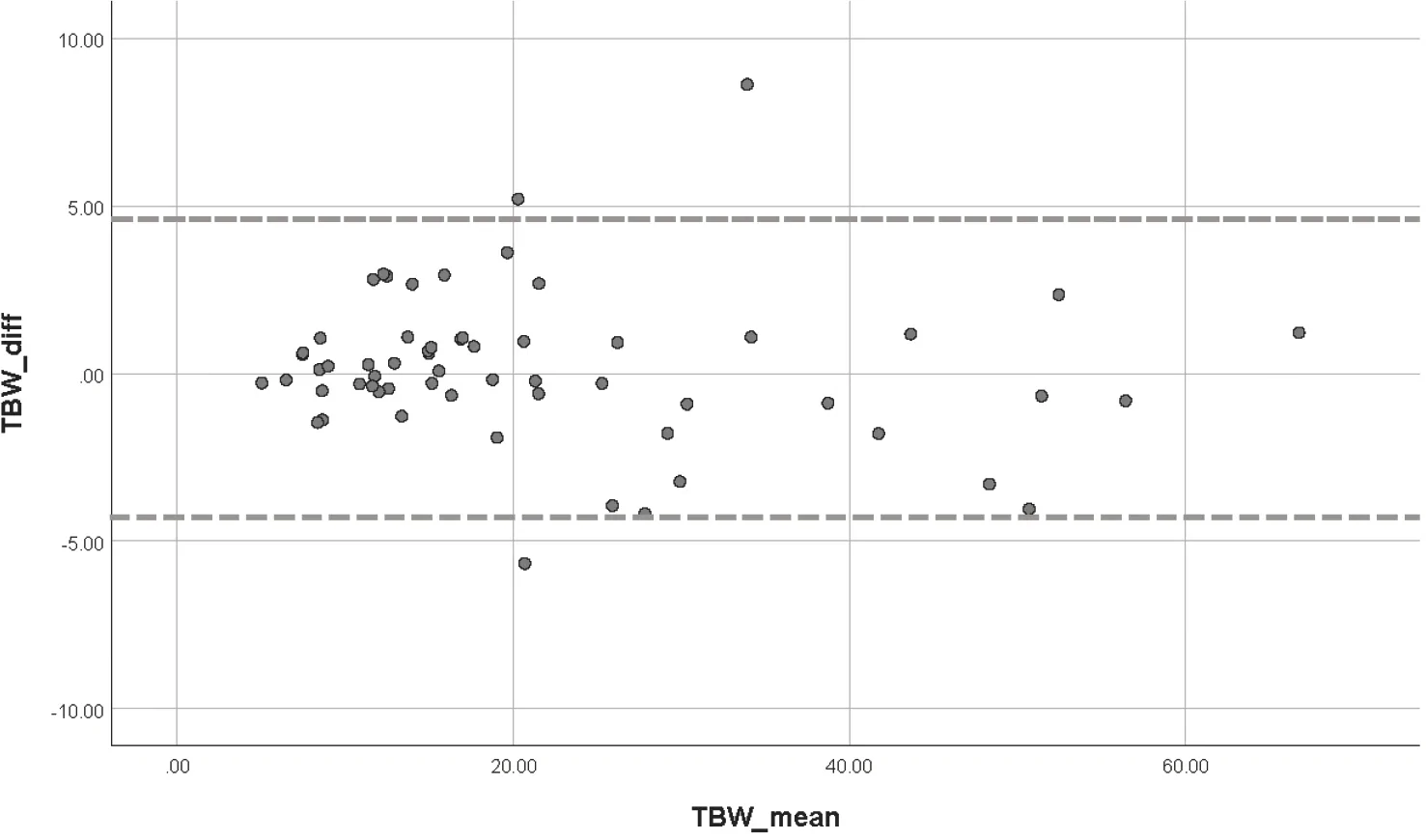
Bland–Altman plot of timed backward walking (TBW) test between Day 1 and Day 2.

### Construct validity

Construct validity was evaluated by examining the relationships between TBW time and other measures using Spearman’s rank correlation analysis. Descriptive statistics for each measure and their correlations with TBW time are presented in Table III.

**Table III:** Correlations between timed backward walking (TBW) time and clinical measures in people with chronic stroke

Outcome measures	People with chronic stroke (*n* = 60)	Spearman’s rho	*p*-value
FMA-LE, median (IQR)	25.00 (7.00)	–0.498	< 0.001*
Muscle strength—ankle dorsiflexor (Affected), kg, median (IQR)	9.50 (6.06)	–0.408	0.001*
Muscle strength—ankle plantarflexor (Affected), kg, mean (SD)	13.39 (6.10)	–0.234	0.072
Muscle strength—ankle dorsiflexor (Unaffected), kg, mean (SD)	14.71 (4.27)	–0.313	0.015*
Muscle strength—ankle plantarflexor (Unaffected), kg, mean (SD)	17.44 (5.27)	–0.108	0.410
BBS, median (IQR)	49.00 (8.00)	–0.647	< 0.001*
LOS, RT, composite, s, median (IQR)	1.20 (0.30)	0.220	0.091
LOS, MVL, composite, deg/s, mean (SD)	2.55 (1.01)	–0.540	< 0.001*
LOS, EPE, composite, %, mean (SD)	44.85 (13.96)	–0.578	< 0.001*
LOS, MXE, composite, %, mean (SD)	57.50 (16.48)	–0.539	< 0.001*
LOS, DCL, composite, %, median (IQR)	59.80 (16.50)	-0.490	< 0.001*
SOT, SOM, mean (SD)	100.55 (3.13)	0.105	0.425
SOT, VIS, median (IQR)	76.50 (15.75)	0.046	0.729
SOT, VEST, mean (SD)	71.65 (12.17)	0.179	0.170
SOT, PREF, median (IQR)	99.00 (6.00)	–0.278	0.032*
10MWT, usual speed, s, median (IQR)	15.27 (7.82)	0.881	< 0.001*
10MWT, max speed, s, median (IQR)	10.92 (6.21)	0.840	< 0.001*
TUG, s, median (IQR)	15.31 (7.67)	0.897	< 0.001*
OX‑PAQ, routine activities, median (IQR)	12.5 (24.11)	0.408	0.001*
OX‑PAQ, social engagement, median (IQR)	0.00 (17.19)	0.174	0.184
OX‑PAQ, emotional well-being, median (IQR)	15.00 (33.75)	0.100	0.448

FMA-LE: Fugl–Meyer Assessment of Lower Extremity; LOS: limits of stability; RT: reaction time; MVL: movement velocity; EPE: endpoint excursion; MXE: max excursion; DCL: directional control; SOT: sensory organization test; SOM: somatosensory ratio; VIS: visual ratio; VEST: vestibular ratio; PREF: preference ratio; BBS: Berg Balance Scale; 10MWT: 10‑metre Walk Test; TUG: Timed Up and Go; OX‑PAQ: Oxford Participation and Activities Questionnaire.

* Correlation is significant at the 0.05 level (2 tailed).

TBW time demonstrated strong, positive correlations with standard timed mobility assessments, such as the TUG test (*ρ* = 0.897, *p* < 0.001) and the 10MWT at both usual (*ρ* = 0.881, *p* < 0.001) and maximum speeds (*ρ* = 0.840, *p*<0.001). Significant negative correlations were observed between TBW times and clinical assessments of balance and motor recovery. Moderate negative correlation was found with the BBS (*ρ* = −0.647, *p* < 0.001) and the FMA-LE (*ρ* = −0.498, *p* < 0.001). Instrumental balance parameters from the LOS test (MVL, EPE, MXE, and DCL) also showed significant moderate negative correlations with TBW time (*ρ* ranging from −0.490 to −0.578, *p* < 0.001). TBW time demonstrated a moderate negative correlation with ankle dorsiflexor strength on the affected side (*ρ* = –0.408, *p*=0.001) and a weak negative correlation on the unaffected side (*ρ* = –0.313, *p* = 0.015). No significant correlations were observed with plantarflexor strength on either side (*p* > 0.05). TBW time correlated moderately with the “Routine Activities” domain of the Ox-PAQ (*ρ* = 0.408, *p* = 0.001), whereas no significant correlations were observed with the “Social Engagement” or “Emotional Well-being” domain (*p* > 0.05).

## DISCUSSION

This study examined the psychometric properties of the TBW test among people with chronic stroke. The TBW test exhibited excellent test–retest, inter-rater, and intra-rater reliability. It showed outstanding discriminative validity, distinguishing people with chronic stroke from healthy adults aged 55 years and older. In addition, the TBW test demonstrated satisfactory construct validity as it significantly correlated with established measures of functional mobility, gait speed, balance, and lower-limb motor function.

### Performance of TBW test in people with stroke

People with chronic stroke took significantly longer on the TBW time than healthy adults aged 55 years and older (*p* < 0.001), supporting the known-groups validity of the test. This difference is likely due to post-stroke impairments, including muscle weakness of the hip extensors, trunk, and other lower limb muscles, which reduce locomotor efficiency, consequently increasing the time to complete the movement (30). Spasticity and abnormal muscle synergies further limit selective joint control, which may further disrupt the coordination required for backward walking (31). Deficits in proprioceptive and vestibular function may also compromise balance and directional control (32, 33). Together, these impairments likely contribute to poorer TBW performance in people with chronic stroke.

The outstanding discriminative ability (AUC = 0.968, 95% CI: 0.934–1.000) indicates that the TBW test can discriminate the performance between stroke survivors and healthy adults using the optimal cut-off of 7.22 s. This benchmark provides clinicians with a rapid and highly sensitive (96.7%) and specific (90.0%) screening tool in community rehabilitation settings. Clinicians working with community-dwelling populations could use this value to identify individuals whose backward walking performance falls below the level expected for neurologically healthy adults.

### Reliability of TBW test in people with stroke

The results demonstrate that the TBW test is a highly reliable and stable assessment tool for individuals with chronic stroke. It demonstrated excellent inter-rater, intra-rater, and test–retest reliability, indicating that it is reproducible across raters and over time. The Bland–Altman analysis showed minimal bias of 0.16 s, indicating that the 1-week test–retest interval successfully attenuated potential practice or learning effects without being confounded by spontaneous neurological recovery. Narrow limits of agreement supported consistent performance across repeated testing on different days under the same testing conditions. In addition, the MDC_95_ of 4.49 s provides a useful clinical benchmark, suggesting that changes greater than this value are likely to reflect real change rather than measurement error.

### Correlations with other outcome measures

Moderate correlations of TBW time with FMA-LE scores and ankle dorsiflexor strength highlight the specific neuromuscular demands of backward walking. Increased tibialis anterior activity of approximately 154% during backward vs forward walking suggests that distal motor recovery may contribute importantly to TBW performance (34). These findings support the TBW test as a useful functional complement to impairment-level assessments.

TBW time was moderately correlated with the BBS and LOS parameters (MVL, EPE, MXE, DCL). Backward walking places greater demands on postural control and dynamic balance than forward walking (3). Better balance, weight-shifting ability, and larger stability limits were associated with faster TBW performance, consistent with previous findings for the 3MBWT (13).

Among the SOT measures, only the visual preference ratio (SOT PREF) showed a significant, weak, negative correlation with TBW time (*ρ* = –0.278). This is because the SOT PREF ratio reflects the ability to suppress inaccurate visual inputs and rely on somatosensory and vestibular cues for postural control (22). This finding suggests that TBW performance is partly influenced by sensory reweighting and the patient’s ability to adapt to environments with restricted or altered visual information during backward walking.

TBW time exhibited excellent convergent validity with standard clinical measures of gait and mobility, such as the TUG test and 10MWT. The strong positive correlations (*ρ* > 0.8) indicate that patients who require more time to complete forward walking and functional turning tasks also perform slower during backward walking. The TBW test may maintain greater discriminative sensitivity among individuals at advanced stages of motor recovery because it imposes greater demands on proprioceptive integration and paretic limb motor control.

Among the 3 domains of the Ox-PAQ, only the routine activities domain demonstrated a significant moderate correlation with TBW time, whereas the social engagement and emotional well-being domains were not significantly associated. This pattern suggests that backward walking performance, as measured by the TBW test, is primarily reflective of functional capacity at the activity level of the International Classification of Functioning, Disability and Health (ICF) framework (35). By contrast, the domains of social participation and emotional well-being may be more influenced by broader environmental factors, such as social policies, community infrastructure, and the availability of social support networks, which exceed the scope of physical performance tests such as the TBW test.

### Strength and clinical implication

The findings of this study carry several practical implications for clinical rehabilitation. Although individuals rarely walk backwards for 10 m continuously in daily life, the 10-m TBW test offers distinct clinical advantages over shorter assessments. It acts as a continuous motor challenge that evaluates steady-state backward gait, sustained dynamic postural control, and endurance. This continuous demand helps unmask subtle residual deficits in proprioception, coordination, and visual dependency that might not be apparent during short bursts of movement, making it particularly valuable for higher-functioning stroke survivors. First, the excellent reliability of the TBW test and its ease of administration make it highly feasible for use in community-based and outpatient rehabilitation settings where access to sophisticated instrumented gait analysis is often limited. Second, the TBW test serves as a valuable adjunct to traditional forward walking assessments. It can help clinicians unmask residual balance deficits and visual dependency that might otherwise go undetected in higher-functioning stroke survivors. Third, the cut-off value of 7.22 s may assist clinicians in identifying stroke-related balance and mobility deficits among community-dwelling older adults presenting to rehabilitation services. Finally, the moderate correlations between TBW performance and specific motor impairments highlight that incorporating backward walking into rehabilitation training programmes could improve these underlying deficits and enhance independence in routine daily activities.

### Limitations

Several limitations of the present study should be acknowledged. First, the cross-sectional design precludes causal inferences regarding the relationship between backward walking performance and clinical outcomes and does not permit evaluation of the responsiveness of the TBW test to rehabilitation-induced change over time. Second, participants were recruited exclusively through community-based self-help rehabilitation groups, which may have introduced a selection bias towards individuals with relatively higher levels of functional independence and motivation, potentially limiting the generalizability of the findings to acute or subacute stroke populations, or to more severely disabled individuals. Third, the present study did not stratify participants by stroke type, lesion location, or time since stroke, factors that may independently influence backward walking performance and the strength of its associations with clinical measures. Finally, other outcome measures that may be related to TBW performance were not included, such as hip and knee muscle strength, lower limb muscle flexibility, and quality of life. Furthermore, it should be noted that the TBW test requires a 12-m unobstructed walkway, including 1-m acceleration and deceleration zones. This spatial requirement may limit its feasibility and application in smaller clinical settings, crowded hospital wards, or patients’ homes, where shorter assessments might be more practical. Future longitudinal studies recruiting more diverse stroke populations and incorporating a broader range of clinical and functional measures would help to address these limitations and further establish the clinical utility of the TBW test.

### Conclusion

The Timed Backward Walk Test demonstrated excellent reliability, outstanding discriminative validity, and satisfactory construct validity in community-dwelling individuals with chronic stroke. The established MDC_95_ of 4.49 s and the optimal cut-off of 7.22 s provide clinically actionable benchmarks for monitoring rehabilitation progress and facilitating community-level screening. Given its ease of administration and strong psychometric properties, the TBW test represents a feasible and clinically meaningful complement to conventional forward gait assessments, particularly for higher-functioning individuals. Future research should investigate the longitudinal responsiveness of the TBW test and its predictive validity for fall risk and long-term functional outcomes.

## Supplementary Material



## Data Availability

The authors confirm that the data supporting the findings of this study are available within the article.
