# Supplementary material for: PSYCHOMETRIC PROPERTIES OF THE TIMED BACKWARD WALK TEST IN PEOPLE WITH STROKE
Source: J Rehabil Med. 2026 Oct 12;58:46292. doi: 10.2340/jrm.v58.46292 (PMC13670358; doi:10.2340/jrm.v58.46292)

Fig. S1. Flowchart of the study.

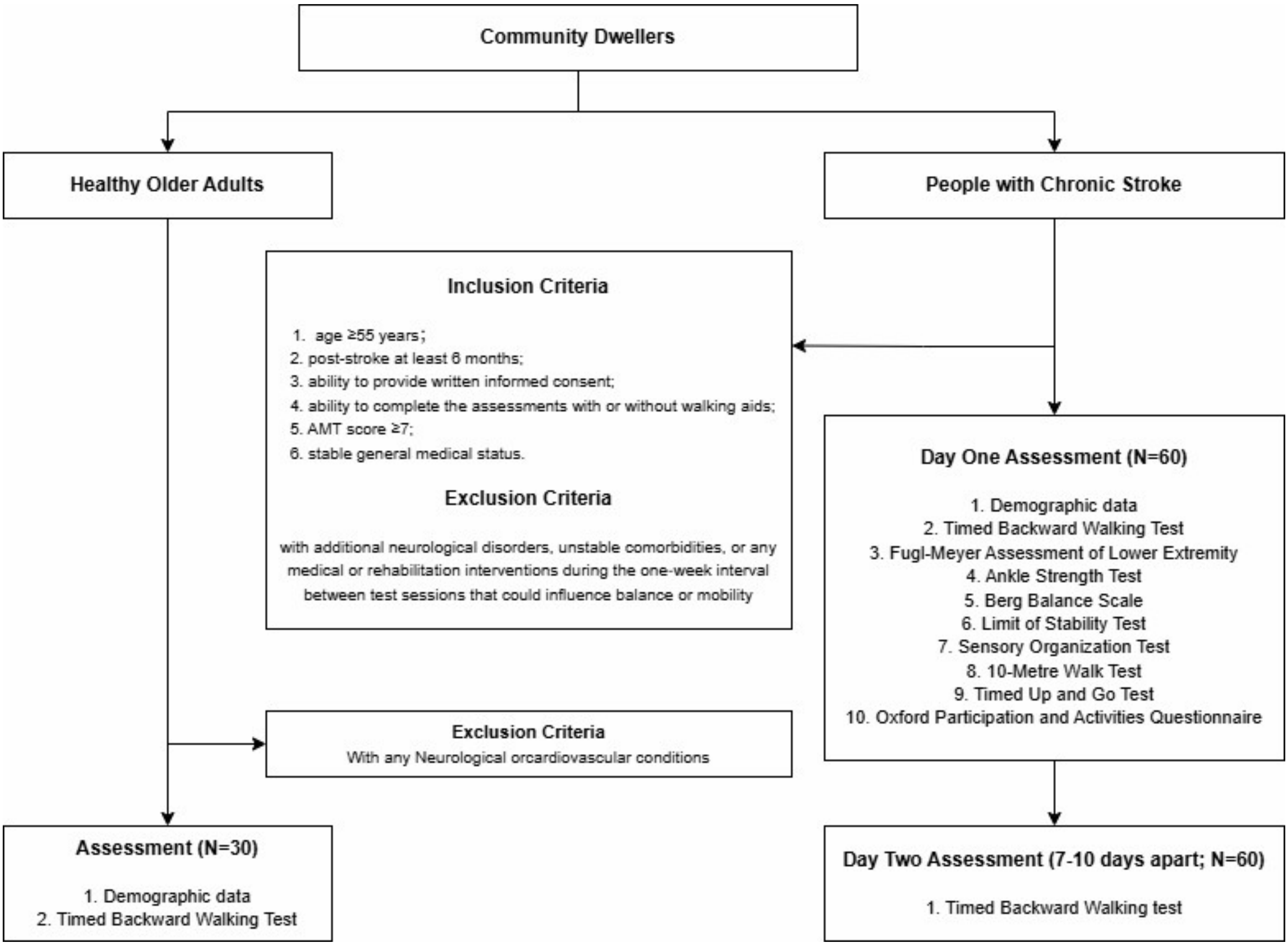

Supplement: Supplementary file 1 [file JRM-58-46292-s1.pdf]
